# Neuroregeneration and plasticity: a review of the physiological mechanisms for achieving functional recovery postinjury

**DOI:** 10.1186/s40779-020-00259-3

**Published:** 2020-06-15

**Authors:** Palaniappan Ganesh Nagappan, Hong Chen, De-Yun Wang

**Affiliations:** 1 School of Clinical Medicine, University of Cambridge, Cambridge, CB2 1TN UK; 2 Shengli Clinical College of Fujian Medical University; Department of Neurology, Fujian Provincial Hospital, Fuzhou, Fujian 350001 China; 3 Department of Otolaryngology, Yong Loo Lin School of Medicine, National University of Singapore, Singapore, 119228 Singapore

**Keywords:** Neuroregeneration, Plasticity, Neuronal systems, Postinjury, Central nervous system, Peripheral nervous system, Rehabilitation

## Abstract

Neuronal networks, especially those in the central nervous system (CNS), evolved to support extensive functional capabilities while ensuring stability. Several physiological “brakes” that maintain the stability of the neuronal networks in a healthy state quickly become a hinderance postinjury. These “brakes” include inhibition from the extracellular environment, intrinsic factors of neurons and the control of neuronal plasticity. There are distinct differences between the neuronal networks in the peripheral nervous system (PNS) and the CNS. Underpinning these differences is the trade-off between reduced functional capabilities with increased adaptability through the formation of new connections and new neurons. The PNS has “facilitators” that stimulate neuroregeneration and plasticity, while the CNS has “brakes” that limit them. By studying how these “facilitators” and “brakes” work and identifying the key processes and molecules involved, we can attempt to apply these theories to the neuronal networks of the CNS to increase its adaptability. The difference in adaptability between the CNS and PNS leads to a difference in neuroregenerative properties and plasticity. Plasticity ensures quick functional recovery of abilities in the short and medium term. Neuroregeneration involves synthesizing new neurons and connections, providing extra resources in the long term to replace those damaged by the injury, and achieving a lasting functional recovery. Therefore, by understanding the factors that affect neuroregeneration and plasticity, we can combine their advantages and develop rehabilitation techniques. Rehabilitation training methods, coordinated with pharmacological interventions and/or electrical stimulation, contributes to a precise, holistic treatment plan that achieves functional recovery from nervous system injuries. Furthermore, these techniques are not limited to limb movement, as other functions lost as a result of brain injury, such as speech, can also be recovered with an appropriate training program.

## Background

According to the Spinal Cord Injury (SCI) Facts and Figures at a Glance, released by the National Spinal Cord Injury Statistical Centre in 2019, there are approximately 17,000 new cases of SCI each year in the United States [1, 2]. Per the Defense and Veterans Brain Injury Center (DVBIC) data, 413,858 individuals within the Department of Defense in the United States sustained a traumatic brain injury (TBI) between 2001 and 2019 [3], with more than one-third having been exposed to a blast event [4, 5]. These two statistics demonstrate the magnitude of the different types of nervous system injuries that veterans are susceptible to. The nervous system comprises the central nervous system (CNS) and the peripheral nervous system (PNS). The CNS consists of the brain and spinal cord, while the PNS consists of cranial and spinal nerves along with their associated ganglia. The PNS has an intrinsic ability for regeneration and repair; however, the CNS is largely unable to self-repair. Moreover, the intrinsic regenerative ability is self-limiting, depending on the characteristics and type of injury, such as those induced by chemotherapy [6].

Current treatment options available after injury to the CNS are limited, often consisting of palliative care [7]. The reasons for these limited options are due to both the intracellular and extracellular factors within the CNS that hinder regeneration. This review investigates the physiological reactions to injuries to the nervous system and attempts by the system to recover to its prior functional state. By comparing the differences in the PNS and CNS, we can help elucidate these mechanisms.

Neuroregeneration and plasticity changes occur first at the regional level in an attempt to revive immediate function and bridge the short-term requirements of the nervous system. While this is occurring, the lengthy process of restoring function with greater permanence occurs at a cellular level. When these processes are combined with rehabilitation techniques [8], a synergistic effect leads to the functional recovery of nervous system injuries sustained in the field.

## Neuroregeneration in the PNS and CNS

### Neuroregeneration in the PNS

**Fig. 1:**
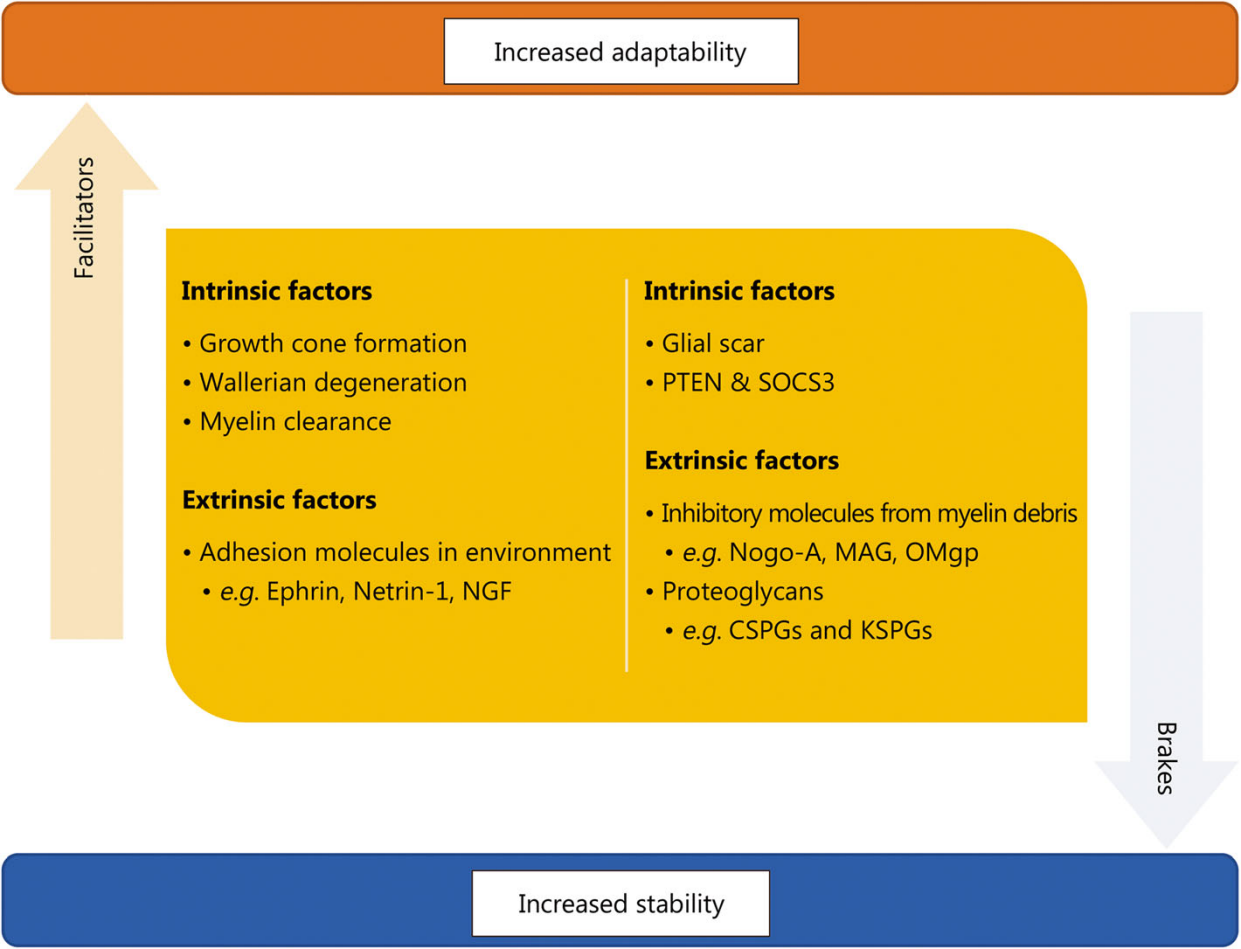
Extrinsic and intrinsic factors that affect neuroregeneration in the central and peripheral nervous systems. PTEN. Phosphatase and tensin homolog; SOCS3. Suppressor of cytokine signaling 3; NGF. Nerve growth factor; MAG. Myelin-associated glycoprotein; Omgp. Oligodendrocyte.myelin glycoprotein; CSPG. Chondroitin sulfate proteoglycans; KSPG. Keratin sulfate proteoglycans

The PNS and CNS have several differences in terms of the balance between “facilitators” and “brakes”, which are illustrated in Fig. 1. The PNS has significant regenerative properties, both morphological and functional. Above and below the lesion, sprouting occurs to make connections, which eventually leads to neuroregeneration. Lesions can result in the activation of otherwise silent connections with ganglia below the lesion site, leading to functional resolution.

**Fig. 2:**
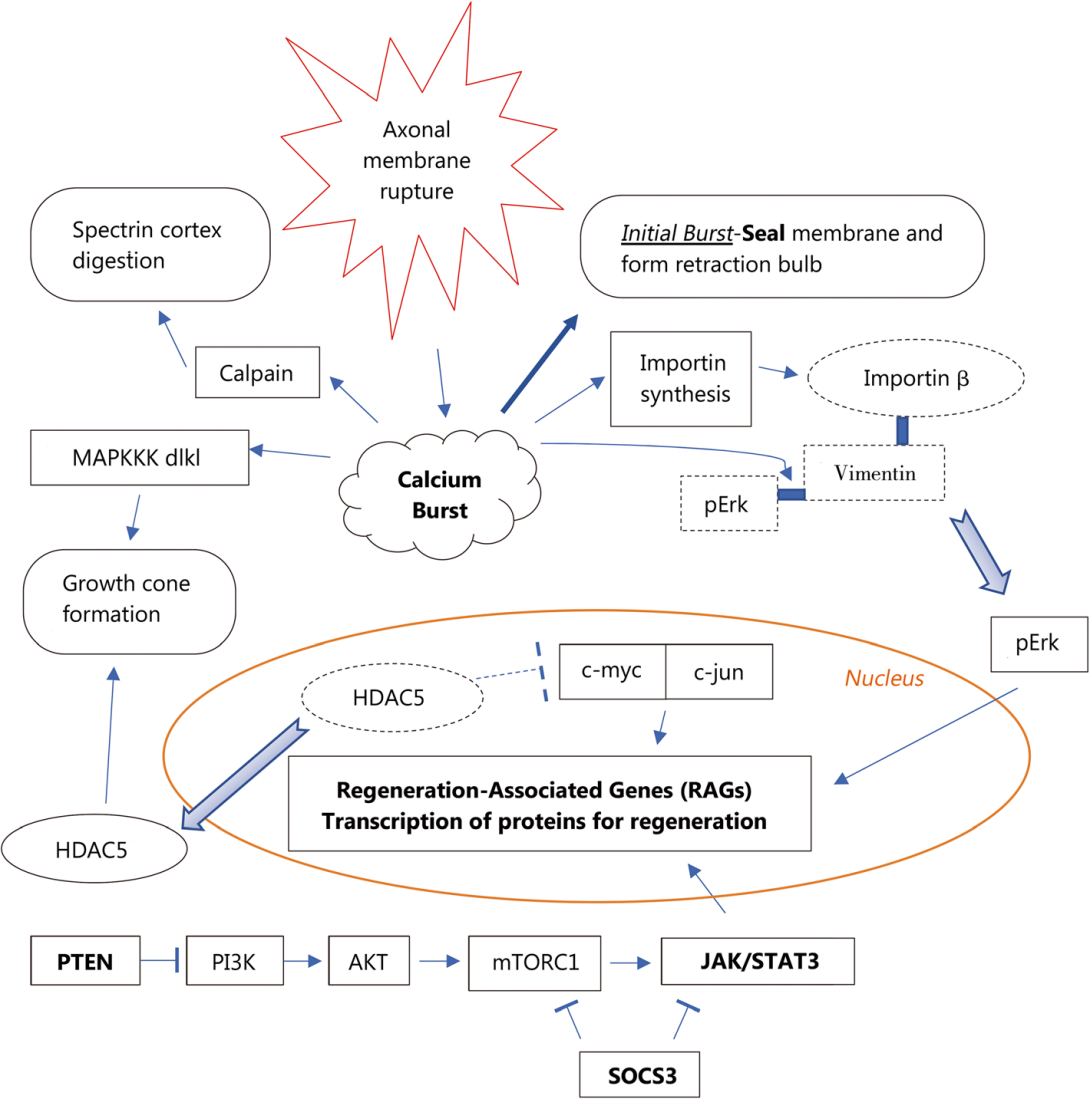
Cascade of reactions from a calcium burst and methods of activating regeneration-associated genes (RAGs). MAPKKK dlk1. Mitogen-activated protein kinase kinase kinase dlk-1; pErk. Phosphorylated extracellular signal-regulated protein kinases; HDAC5. Histone Deacetylase 5; RAGs. Regeneration associated genes; PTEN. Phosphatase and tensin homolog; PI3K. Phosphoinositide 3-kinases; AKT. Protein kinase B; mTORC1. Mammalian target of rapamycin complex 1 or mechanistic target of rapamycin complex 1; SOCS3. Suppressor of cytokine signaling 3; JAK/STAT 3. Janus kinase/signal transducer and activator of transcription 3

The initial injury leads to acute axonal degeneration (AAD), causing the distal and proximal ends to separate within 30 min of the injury [9]*.* This is a crucial process initiated by the initial influx of calcium [10], as seen in Fig. 2, that begins the entire process of degeneration through the preliminary clearing of the damaged parts of an axon. Dystrophic bulb structures then begin to form at both terminals while the membranes are sealed. Following this formation, sprouting has been observed to occur, which forms the growth cone. Upon contact with adhesion molecules in the environment, the growth cone then orientates towards the regions with adhesion molecules [11]. This steering of the growth cones towards areas with high concentrations of adhesion molecules provides a suitable means of reconnecting both axonal ends. Adhesion molecules can be either membrane-bound (e.g., Ephrin and Semaphorin) or diffusible factors (e.g., Sema3A, NGF, Netrin-1, Reelin and Slit) [12, 13]. Growth cones derive their building materials from 4 sources: the environment; transport vesicles initially localizing to axon terminals; mRNAs translated locally to synthesize the proteins; and recycled axonal molecules such as actin and tubulin [14].

As a result of injury to the axon, axonal permeability to calcium is temporarily increased, lasting minutes. This prolonged access creates a high-concentration calcium pulse that activates several factors, including calpains [15], as shown in Fig. 2. The initial part of the calcium influx enables the axon to seal itself and form a retraction bulb, while the later part of the influx activates calpains that digest the submembranous spectrin cortex. This digestion facilitates successful regeneration of the axon post-injury as it provides access for transport vesicles to reach the surface of the axon tip to deposit new receptors and membrane components. Without this access, these transport vesicles would accumulate within the retraction bulb, resulting in a static end bulb [16]. The other key signaling pathway activated by the calcium pulse is MapKKK dlk-1, which is essential for the formation of growth cones and thus regeneration [17], as shown in Fig. 2.

To sustain the development of growth cones, large-scale protein synthesis needs to occur. The axons themselves contain approximately 3000 mRNAs, which are specific for axonal maintenance, repair and regeneration. The axons also contain ribosomes and Golgi-like structures that produce proteins locally [18]. In addition, calcium influx induces several important effects. It upregulates the translation of importins and RanBP, which is transported through retrograde action, picking up Vimentin fragments and Erk and inducing their transport to the nucleus to trigger the expression of regeneration-associated genes (RAGs) [19]. The calcium pulse also causes a cytoplasmic shift of HDAC5 to leave the nucleus via PKCμ. This translocation allows for previously suppressed genes to be activated via acetylation of histones, stimulating the synthesis of RAG-associated transcription factors such as c-jun and c-fos. HDAC5, as a result of its cytoplasmic translocation, is transported to the growth cone, where it helps to promote axon growth via microtubule deacetylation [20]. Furthermore, RAGs facilitate axon priming, which is known as a conditioning effect, for faster regeneration in the event of future injuries. These are some of the key aspects of the post-injury status that occurs within an axon to support growth cone formation and nerve regeneration in the axons proximal to the lesion.

Modifications occur in the axon distal site prior to reconnection with the proximal end. The axon beyond the lesion rapidly degenerates in an active process – Wallerian degeneration – and is triggered by depletion of the rapidly degrading NMNAT2 as controlled by SARM1 [21, 22]. Within 48 h, the myelin sheaths begin to separate at the Schimdt-Lanterman incisures before forming bead-like structures. Initially, Schwann cells phagocytose myelin debris [23] while waiting for macrophages to respond to secreted cytokines and chemokines. At the three-week point after the injury, the influx of macrophages reaches its peak, clearing the remaining debris using opsonins, complement antibodies, and pentraxins [24]. The purpose of this influx could be to generate a blank slate upon which regeneration can begin without interference from fragments of older material, thus facilitating neuroregeneration.

Once the myelin debris from the distal axons have been cleared, the process of regeneration can begin as the “brakes” have been lifted. The expression of nerve growth factor (NGF) mRNA is upregulated five- to seven-fold within a period of 2 weeks. This increase can be attributed directly to the effects of nerve fibroblasts and Schwann cells [25] and indirectly to macrophages that stimulate these cells with macrophage-derived interleukin-1 [26]. Other neurotrophic factors released include brain-derived growth factor, insulin-like growth factor, fibroblast growth factor, NT3, artemin and glial cell line-derived neurotrophic factor. Damage-related signaling molecules, such as p38 MAPK, activate the transcription factor c-jun, which in turn induces Schwann cells to change into specialized repair cells – Bands of Bungner within the basal laminar tube [27]; these cells provide structural guidance to further enhance regeneration, without which regeneration would not be possible [28].

A time limit has been observed in several cases, proving that regeneration can occur only within a narrow window after injury. Regeneration proceeds in the PNS at 1 mm per day and can bridge gaps of 1 cm. Within 2–3 months, Schwann cells lose their permissiveness to regeneration signaling. Thus, for a long limb, such as the arm or leg, only the proximal limb may be reinnervated, with the chances for distal reinnervation dropping drastically [29, 30]. Notably, the muscle cells need not be at the muscle endplate for reinnervation. Only the endplate extracellular matrix containing S-laminin and agrin, the remaining Schwann cells and capping cells are required to provide guidance for reinnervation [31].

It is a clinically observable fact that the regenerative capacity of the nervous system declines with age, corresponding to the residual functional reserve of individuals. Painter and colleagues replicated this phenomenon in aged mice. However, they found that it was not the loss of regenerative ability of the axons that caused the decline; instead, it was the decline of glial function that caused the loss. Ageing glia results in the slow clearance of myelin debris, causing slower Wallerian degeneration. This impaired Wallerian degeneration can thus lead to an overall impaired regenerative capacity of the nervous system [32, 33].

The high influx of calcium as described above and in Fig. 2, left unchecked, leads to the activation of hydrolytic enzymes, exaggerated energy expenditure and impaired energy production, eventually resulting in cell death. This disruption in mitochondrial dynamics has been implicated in behavioral impairment and cognitive deficits [34]. In the postinjury state, large amounts of energy are required for the nerve to return to and be maintained at its homeostatic point, with even greater amounts needed for its self-regeneration. Therefore, supplementing the injured nerve with freshly isolated mitochondria provides a means to resolve the cellular energy crisis and facilitate regeneration [35, 36].

### Neuroregeneration in the CNS

CNS injuries have been known to lead to poor prognoses because of their inability to regenerate neurons, in contrast to the response to PNS injuries [37]. Furthermore, this distinction is not seen in all species. Rodents can restore myelin sheaths to almost all demyelinated axons [38], and zebrafish are able to efficiently regenerate the spinal cord [39, 40]. Why has it been evolutionarily beneficial for the human CNS to not regenerate? This state could be a result of the high complexity of the neuronal networks within the human CNS compared to that of other species. Whereby adding further neurons to the already intricate neuronal networks would be deleterious, as it would risk causing confusion to the system by generating foci of inappropriate electrical activity, similar to short-circuiting an electronic device, thereby increasing the likelihood of seizures. When the brain is healthy, this rigidity and constancy enables the maintenance of normal function. However, under the burden of disease and injury, these limits become obstructive to the treatment of patients. To understand how the CNS can be forced to regenerate, we first must look at the initial causes of the impairment to this process.

Neuroregeneration in the CNS is turned off because of the lack of intrinsic CNS axon regeneration ability and extrinsic inhibition conferred by the CNS environment.

#### Lack of intrinsic regenerative ability of CNS axons

CNS axons lose their regenerative ability during development. Embryonic axons have been found to have a much greater ability to grow in the CNS than adult axons. Embryonic neurons implanted into the adult CNS can grow extensively despite the inhibitory environment. Furthermore, embryonic spinal cord precursors have been shown to accept input from the host axons to fully integrate with the adult cord, acting as a form of relay [41]. This finding demonstrates that an answer for neuroregeneration that overcomes the intrinsic inability of adult neurons to regenerate lies within the expression of neuronal genes.

PTEN and SOCS3 are proteins that have been found to play roles in inactivating regeneration in CNS neurons by inhibiting AKT and JAK/STAT signaling, respectively, as shown in Fig. 2; these two signaling pathways play roles in promoting survival and growth. The concurrent inactivation of PTEN and SOCS3 results in the coactivation of specific gene transcription and protein translation that permits profuse regeneration of the optic nerve and moderate regeneration of the spinal cord [42, 43]. Importantly, this regeneration does not induce CNS neurons into a PNS-like state, as PTEN is similarly expressed and SOCS3 levels are increased in PNS neurons during regeneration [44, 45]. Nevertheless, increasing mTOR activity via the deletion of PTEN and SOCS3 enhances axonal regrowth in PNS neurons [45, 46]. This finding indicates that the method by which the deletion of PTEN and SOCS3 acts may involve different growth suppressive mechanisms. Given the inhibitory environment within the CNS, the synergistic effects of inhibiting these two different pathways remain avenues for inducing neuroregeneration within the CNS [47, 48].

The axons contain only a subset of the molecules present in cell bodies. Without the necessary molecules, regeneration is impaired. Some of the critical molecules that are absent include integrins, several different growth factor receptors and ribosomes. Integrins contribute to the reorganization of extracellular matrix glycoproteins, while growth factor receptors respond to growth factors in the environment. In the injured CNS environment, tenascin, an extracellular matrix glycoprotein, is greatly upregulated after CNS injury. Unfortunately, adult CNS neurons do not express a tenascin-binding integrin. Promising results were observed when α9β_1_ tenascin-binding integrin combined with the β_1_-binding integrin activator kindlin-1 was expressed in crushed dorsal root ganglia via transgenic adeno-associated viruses. Kindlin-1 was necessary to attenuate the inhibition of chondroitin sulfate proteoglycans (CSPG). Twelve weeks after the crush injury, the axons grew from the C6–7 level to a level above C1, covering a distance of more than 25 mm and 7 spinal levels via the normal pathway. Further anatomical and electrophysiological analyses demonstrated that the connections within the spinal cord were topographically correct. Recovery in response to mechanical pressure, thermal pain and ladder-walking tasks was observed [49].

In addition to the reduction or inhibition of gene expression during CNS axon maturation, the lack of certain molecules in the axons can be due to impaired transport mechanisms to the site of injury. Integrins virally expressed in the adult rat sensorimotor cortex and adult red nucleus, but not in the dorsal root ganglia, did not localize to axons. However, when expressed in developing rat cortex (postnatal day 5 or 10), clear localization was observed in the axons of the corpus callosum and internal capsule. In newborn rodent CNS neurons, integrin permissively travels down the corticospinal tract; however, in adult rodents, integrin transport does not continue past the initial segment, limiting its function [50]. Therefore, there is a differential ability in the axonal transport of transmembrane proteins in vivo, which is dependent on the subtype of the neurons and their age.

**Fig. 3:**
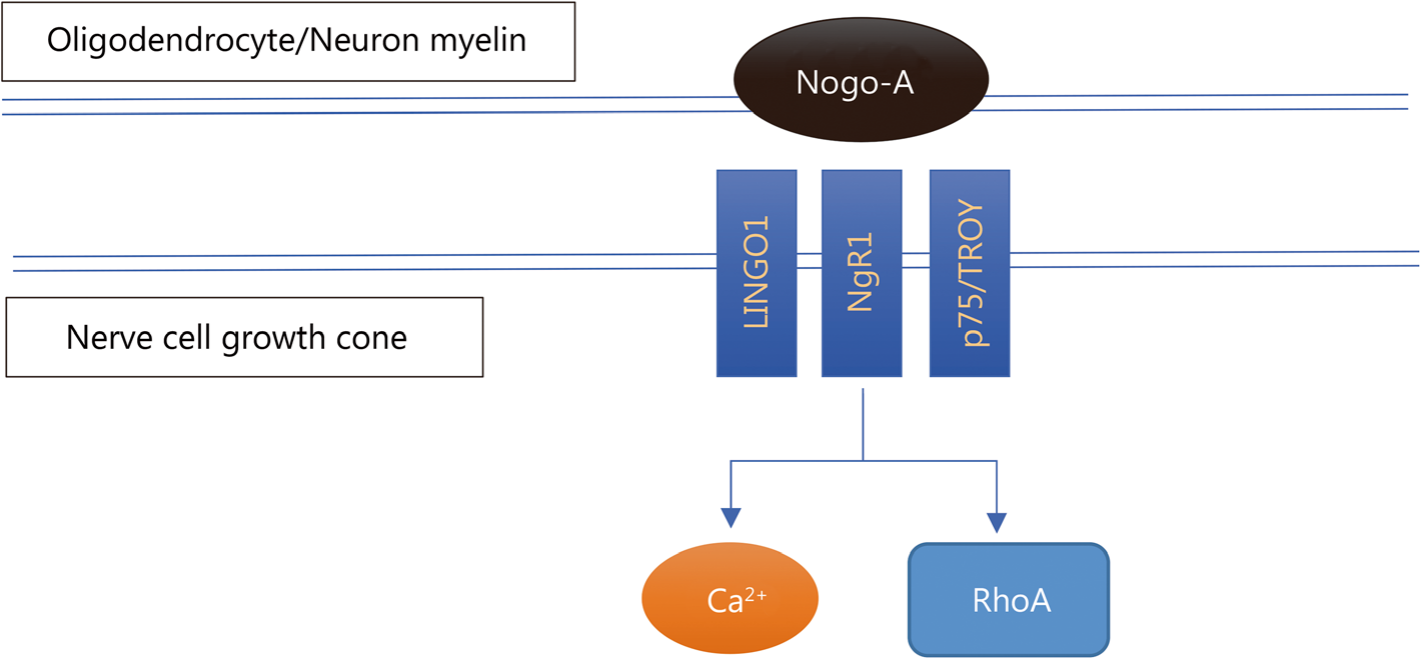
Nogo-A mechanism of action. Nogo-A interacts with several receptors, the most important of which are NgR1, LINGO1 and p75/TROY. This interaction creates a cascade that inhibits neuroregeneration in the nerve cell growth cones. LINGO1. Leucine rich repeat and Immunoglobin-like domain-containing protein 1; p75. Neurotrophin receptor; TROY. Tumor necrosis factor receptor superfamily, member 19; RhoA. Ras homolog family member A

Another method of enhancing intrinsic regenerative ability is to remove any inhibition acting on the pathway. Many of the CNS inhibitory effects are relayed through RhoA; an example is shown in Fig. 3. Inactivating RhoA with the *Clostridium botulinum* C3 ribosylating exoenzyme has been shown [51]. Based on studies on damaged rodent spinal cords with BA210 (cethrin) [52], a modified inhibitor able to penetrate the dura and cell membrane, phase I/II clinical trials with 48 patients demonstrated profound neurological recovery in patients with cervical spinal cord injury and only modest recovery in those with thoracic spinal cord injury [53–55].

#### Extrinsic inhibition by the CNS environment

When a CNS axon is placed outside its typical setting, such as in the permissive environment of a peripheral nerve, regeneration can be observed to an extent [56]. However, this effect is limited to regeneration in the permissive environment of a PNS graft, as it does not extend to the CNS tissue [57]. This demonstrates that there are factors within the CNS environment that prevent neuroregeneration, and they are attributed to inhibition induced by glial scars and myelin oligodendrocytes.

1. Glial scar

Myelin in the CNS is largely derived from oligodendrocytes, in contrast to myelin in the PNS, where it is derived from Schwann cells. A key difference of Schwann cells and oligodendrocytes is that the latter require axon signals to survive. In the CNS, space and resources are limited; thus, during development, only the oligodendrocytes that make contact with axons and receive axonal signals survive, while the others undergo apoptosis. This characteristic is carried forward into maturity and contributes to the inhibition observed.

Therefore, upon injury, axonal signals are lost, resulting in the oligodendrocytes undergoing either programmed cell death or senescence. As a result, the myelin sheaths remain, and their debris is not cleared. The duration of this process was observed to be as long as 22 months in rats [58]. As a result, regeneration of the CNS is hindered due to the lack of clearance. Eventually, a reactive cellular process, involving an abnormal increase in astrocytes (astrogliosis), forms glial scars, further hindering the chances of regeneration and reinnervation.

Another contributing factor to glial scar formation is the lack of myelin clearance by macrophages and microglia in the CNS. Microglia are the resident macrophages of the CNS and comprise 10–15% of the cells in the CNS [59], and therefore, they are also called upon to clear the myelin from the distal parts of the injured axons. However, compared to the process in the PNS, recruitment of these microglia to the injury site takes 3 days longer. Of those microglia that arrive at the lesion, only a fraction are transformed to effectively clear the debris [60]. Furthermore, the clearance rates of microglia are lower than those of macrophages. This difference can be attributed to the lack of opsonin activity around microglia and the low permeability of the blood-brain barrier (BBB), which hinders macrophage infiltration [23].

The question remains whether glial scar formation is a byproduct of pathological activity in the CNS or whether it serves an evolutionary purpose in the maintenance of a healthy brain. Glial scars consist of reactive astrocytes as the main component along with microglia, endothelial cells, fibroblasts and a basal membrane [61]. They prevent neuronal regeneration by forming a chemical and physical barrier to axonal extension. Despite creating this barrier, they have a role in the revascularization of blood capillaries to provide trophic, nutritional and metabolic support to nerve tissue, with an ultimate function of re-establishing the chemical and physical integrity of the CNS. The absence of the glial scar is associated with problems in the repair of the BBB [62]. Temporarily removing CNS glia provides a tunnel through which axons can extend and regenerate temporarily. Removing astrocytes in the glial scar or the glial scar itself can cause damaging inflammation, failed BBB [63] re-formation and problems in the repair of the BBB [62].

Thus, being able to selectively turn off glial scars and inhibitory signals in the extracellular matrix would be useful in neuroregeneration.

Astrocytes constitute a key cell type recognized for inhibiting axonal regeneration. However, there is great heterogeneity among astrocytes – comprising both permissive and inhibitory astrocyte subpopulations. A comparison of these two types showed that the inhibitory molecules are chondroitin sulfate proteoglycans (CSPGs), which are upregulated after injury [64, 65]. The repeating disaccharides of glucuronic and galactosamine, glycosaminoglycans (CS-GAGs), are covalently coupled to the protein core of CSPGs and are thought to be the main inhibitory part of a CSPG. By digesting this chain with chondroitinase ABC, CSPG inhibition can be suppressed [66]. Axonal regeneration and a reduced inhibition barrier between the CNS tissue and nerve grafts were observed upon injection of chondroitinase ABC into the CNS. Chondroitinase ABC has also been shown to improve recovery from spinal cord injuries [66] when combined with other techniques, such as nerve guidance conduits, Schwann cell transplants [67] or peripheral nerve autografts [68].

Similar to CSPGs, keratan sulfate proteoglycans (KSPGs) have N-acetylglucosamine 6-O-sulfotransferase-1 instead of CS-GAGs. Depletion of KSPGs was also found to suppress the inhibition of nerve regeneration [69].

(2) Myelin

Myelin produced by oligodendrocytes consist of several proteins that influence neuroregeneration, each of which has its own functions. In terms of regeneration, the NOGO (NI-250) family is key, particularly Nogo-A [70]. Nogo-A is involved in autoimmune-mediated demyelination, which includes multiple sclerosis (MS) and experimental autoimmune encephalomyelitis (EAE). Nogo-A can interact with neurons via two main termini: the amino-Nogo terminus via an unknown receptor and the Nogo-66 terminus through NgR1, p75, TROY or LINGO1, as shown in Fig. 3. Remyelination is observed when this inhibitor is antagonized, playing a major role in the RhoA pathway [71, 72]. The Nogo receptor, to which the Nogo-66 terminus binds, is also a receptor of myelin-associated glycoprotein (MAG) and oligodendrocyte myelin glycoprotein (OMgp). Treatment with anti-Nogo-A antibody closely followed by multimodal rehabilitation training showed greater improvements in functional recovery than either method alone [73].

As promising as inhibiting NOGO, MAG or OMgp may be, there have been issues in terms of our understanding of their biology. Nogo-A knockouts or triple knockouts (Nogo-A, MAG and OMgp) both modulate axonal sprouting; however, they fail to exhibit enhanced regeneration of axons in the injured spinal cord. Therefore, they may not play a central role in the failure of regeneration but instead serve in an accessory or contributing role [74].

The current issue may be that we are looking for a silver bullet to treat a central problem in the failure of regeneration, but given the intricacies of the brain, fine modulation of several contributory factors may be necessary to maintain existing brain function while promoting the regrowth of the axons and thus facilitate neuroregeneration.

Notable inhibitory proteins in the CNS are listed in Table 1.

**Table 1:** Proteins in the CNS extracellular matrix that contribute to the inhibition of neuroregeneration after injury

Inhibitory protein	Function	Complementary receptors
Nogo-A	Remyelination inhibitor via the RhoA pathway	Nogo-66 terminus: NgR1, p75, TROY and LINGO1 Amino-Nogo terminus: unknown
MAG	Remyelination inhibitor via the RhoA pathway	NgR2, GT1b, NgR1, p75, TROY and LINGO1
OMgp	Remyelination inhibitor via the RhoA pathway	NgR1
Versican (CSPG2)	Important during inflammation as it interacts with inflammatory leukocytes and inflammatory cells recruiting chemokines. It also stabilizes perineuronal nets to stabilize synaptic connections.	N-terminus: hyaluronan in the extracellular matrix (ECM) C-terminus: Ligands in ECM, especially tenascin [75]
NI-35	Nonpermissive growth factor in myelin	Unknown
Ephrin B3 [71]	Inhibits remyelination	EphA4
Semaphorin 4D (Sema 4D) [71]	Inhibits remyelination	PlexinB1
Semaphorin 3A (Sema 3A) [76–78]	In scars in both PNS and CNS injuries	Nrp1, Nrp2, L1cam, Nrcam [79]

*NgR1* Neuronal Nogo-66 receptor 1, *LINGO1* Leucine rich repeat and Immunoglobin-like domain-containing protein 1, *p75* neurotrophin receptor, *TROY* Tumor necrosis factor receptor superfamily, member 19, *RhoA* Ras homolog family member A, *MAG* Myelin-associated glycoprotein, *GT1b* Trisialoganglioside protein, *OMgp* Oligodendrocyte-myelin glycoprotein, *CSPG2* chondroitin sulfate proteoglycan core protein 2 or versican, *ECM* extracellular matix, *NI-35* A CNS myelin-associated neurite growth inhibitor, *EphA4* Ephrin type-A receptor 4, *Nrp1* Neuropilin 1, *Nrp2* Neuropilin 2, *L1cam* L1 cell adhesion molecule, *Nrcam* Neuronal cell adhesion molecule

## Plasticity in the CNS postinjury

Plasticity in the nervous system optimizes neural networks during ontogeny, phylogenesis, physiological learning and brain injury. In the context of brain injury, we propose that neuroplasticity primarily takes the form of cortical remapping. The functions lost as a result of brain damage can be recovered when the damaged cortex is remapped to another part of the cortex [80]. This remapping is achieved through the generation of new circuits that bypass lesions and restore function. There are three factors that contribute to this process: first, distance between the sprouting of damaged and undamaged axons, with a shorter separation being advantageous; second, modulation of the existing synaptic strengths; and third, alteration of the GABAergic interneuron circuitry. The latter two factors can be induced via metaplastic changes that facilitate and regulate long-term potentiation (LTP) and depression (LTD). When coupled with rehabilitation, these processes can lead to functional recovery of the nervous system.

In healthy people, plasticity occurs constantly to enable memory formation and to cope with functional demands [81]. When rats are trained to retrieve a food pellet, their distal forelimb motor cortex is enlarged at the expense of their proximal forelimb motor cortex [82]. In squirrel monkeys trained to handle small objects the finger regions are enlarged at the expense of their wrist and forearm regions [83]. However, when wrist training replaced the finger training regime, the wrist regions were enlarged, while the finger regions were reduced [84]. Therefore, the adult brain is plastic. After injuries such as strokes and TBIs, specific areas of tissue are lost that compromise the individual’s ability to carry out certain functions. The brain thus needs to reprioritize the functional needs that are important to the survival and well-being of the individual. The cortex is remapped such that the less important functions are diminished as the important functions are enhanced. This cortical remapping occurs alongside the growth of new connections, which is further enhanced by rehabilitation leading to improved functional recovery [85]. In the initial phases after the injury, large areas of the brain have been found to carry out the function of the damaged area. Over time, this area becomes focused towards a single region as a result of cortical remapping [86]. This enables some functional recovery both in the short and medium term.

The mechanism underpinning cortical remapping can be attributed to various forms of functional and structural neuroplasticity, importantly metaplasticity [87–91]. Metaplasticity ensures that synapses are maintained within a dynamic range of plasticity [92]. This is realized by preventing synapses from becoming too strong or weak to prevent excessive or insufficient excitation, respectively. Based on the Bienenstock, Cooper, and Munro (BCM) computation model, the LTP and LTD thresholds are dynamically adjusted based on time-averaged postsynaptic activity. Metaplasticity works on a longer timescale than neuromodulation, which involves neurotransmitters, cytokines and hormones present *at the time* of plasticity induction. It may be the process by which large areas of the brain are initially used to carry out the function(s) of the damaged area(s) before becoming focused on a smaller area. Metaplasticity initially increases the levels of plasticity throughout the brain to enable the initial restoration of function, while a long-term solution is being mapped out at specific regions of the brain through localized regions of plasticity for particular functions. Furthermore, intrinsic plasticity is thought to cooperatively function with synaptic plasticity to cope with damaged areas of the neuronal network [91]. The extent to which intrinsic plasticity regulates neuronal recovery remains a question (e.g., gain, firing rate of neurons, internal calcium concentration), but functionally, it may play a role in learning and represent the intensity of the stimuli in terms of their excitability levels [93]. Thus, in the damaged brain, this mechanism can help alleviate the effects of brain damage by bridging the disparity in firing rates (as a result of missing parts of the neuronal circuit) through modifications of intrinsic excitability [94].

After a spinal cord hemi section in adult rats, bypass circuits were generated within the spinal cord and brain. Although fine motor control of the damaged regions was observed, gross motor control of basic locomotor function saw substantial recovery as a result of this plasticity. Once again, this demonstrates neuroplasticity’s important role in the short-term functional recovery of an individual after injury [95–97].

A crucial aspect that contributed to the functional recoveries described above is the rehabilitation process. Without rehabilitation, the new connections arising from the increased plasticity would serve little purpose. Rehabilitation is the crucial process that provides the necessary stimuli to guide these newly formed circuits into maturity. Importantly, the type of functional recovery is dependent on the type of rehabilitation during the period of increased plasticity. Only the function(s) that are addressed during rehabilitation showed increased recovery, while the functions not addressed during rehabilitation showed poor recovery [98]. Furthermore, this rehabilitation process needs to occur immediately after the initiation of plasticity and not concurrent with it for the appropriate functional recovery [99].

Therefore, being able to reactivate plasticity in the adult brain can be very useful in improving functional recovery and neuroregeneration. Physiologically, there is a period in the human life span in which the brain is highly plastic, allowing it to be actively shaped in an experience-dependent manner. This is termed the critical period. This period lasts in humans from shortly after birth to 5 years of age. Children with brain injuries demonstrate remarkable recovery in motor skills; however, this is at the expense of cognitive ability, as the regions critical for cognition were replaced by those focused on sensorimotor skills [100]. Reactivation of these windows has implications not only in terms of the focal damage caused by brain injuries but also in the context of neurodegenerative disorders such as Alzheimer’s disease (AD), for which a model has shown memory restoration to an extent [101].

Understanding how plasticity is actuated is important in the reactivation during critical period windows. In a cut sciatic nerve, input to the denervated region of the motor cortex can be recorded hours later. This finding indicates that previously suppressed nerves are somewhat activated. It has been postulated that, in addition to normal actively functional nerves, there are intracortical connections suppressed by GABAergic inhibitory circuits. These inhibitory circuits modulate and readjust the motor cortex representations based on the demands and stressors in place [102]. Therefore, the first step in short-term functional recovery would be the removal or modulation of these inhibitory circuits such that new circuits can be stimulated to compensate for the lost functions, thus providing a form of circuitry regeneration.

GABAergic circuits also play a crucial role in creating the precisely timed generation of action potential, which is achieved because the average excitatory and inhibitory signals are similar but not equal [103, 104]. In a long timescale, inhibitory and excitatory signals appear to track one another, and an alteration in one leads to an alteration in the other, producing a stable modification of the firing characteristics of the neuron [105]. Coordinating these alterations in activity at the subcellular, cellular and circuit levels is a set of GABAergic interneurons [106, 107].

In the medium term, new connections need to be developed to replace the damaged circuits. This replacement can be done on the dendritic scale by inducing the generation of new connections between neurons. The formation of dendritic spines, which are membrane projections of dendrites, enable the formation of new connections between neurons, stimulating learning to restore function [108]. Dendritic spine dynamics can store synaptic information because of their great variety of shapes, sizes and numbers [109]. Orlando found that the removal of CSPGs with chondroitinase enhanced spine dynamics, thus inducing plasticity on the dendritic scale [110]. This experiment demonstrated that CSPG plays a significant role in stabilizing neuronal networks and reducing plasticity.

Later, chondroitinase was been found to also digest a stabilizing perineuronal net that encases the GABAergic circuitry during the maturation of neurons. The formation of this perineuronal net (PNN) coincides with the end of the critical period of plasticity [111]. The PNN is mainly composed of hyaluronan, CSPG, link proteins and tenascin R. The protein link between CSPG and PNN is the key contributor to the plasticity inhibition induced by PNN [112]. Furthermore, the diffusible transcription factor Otx2 has been found to be transported from the eye, promoting the onset of critical period plasticity [113]. After amblyopic subjects played an action video game, an increase in plasticity was observed [114].

The purpose of the PNN is to restrict the formation of additional connections to the GABAergic neuronal network. In a healthy adult, this restriction serves to maintain a stable set of parameters within the neuronal circuits for normal daily functions. However, during injury, it becomes a hinderance as the neuronal circuits are still fixed but with a portion of the network being damaged. This situation leads to the neurological and psychological deficits observed. However, being able to add new connections and modulate existing connections in the inhibitory GABAergic network would instate the ability of existing cortical maps to reorganize themselves based on the demands exerted on the neuronal network, thus enabling plasticity and providing a method for functional recovery in the short and medium term.

## Rehabilitation

Studies on neurorehabilitation began after the two world wars to help treat brain-injured soldiers. There are two main types of injuries sustained to the nervous system: gunshot wounds and blast injuries. The injury profiles of these two injuries are distinct. Gunshot wounds generally lead to more focal damage associated with SCI and focal injuries in the brain. However, blast injuries and blunt trauma [115] lead to more diffuse damage, such as diffuse axonal injuries, or affect large areas within the brain [116]. Therefore, functional recovery postinjury is highly dependent on the mechanism of injury and treatment of the damaged tissue accordingly. In the most severe cases, such as in diffuse axonal injury, there is widespread damage throughout the brain, resulting in minimal functional tissue for the continued survival of the individual. However, in many cases, there is still sufficient tissue for survival. However, the quality of life in these cases is often poor, as certain functions are lost, depending on the extent of the brain damage, ranging from paraplegia to anomia. Plasticity and neuroregeneration alone are insufficient to achieve significant functional recovery. Rehabilitation plays a key role in ensuring that the potential generated by plasticity and neuroregeneration can be guided to restore lost functions.

As described above, when rehabilitation is conducted after plasticity is induced, recovery of the rehabilitated function is improved. However, there are barriers to translation when such rehabilitation is conducted in humans as opposed to nonhuman mammals. In addition to rehabilitation, humans require greater supraspinal input to achieve outcomes similar to those of nonhuman mammals, indicating the greater reliance of the spinal cord on the brain [117]. In SCI, this supraspinal input is often affected by denervation; however, when plasticity and axonal regeneration are induced, detour circuits can be formed through other neuronal tracts, such as the propriospinal tract, within the spinal cord. Therefore, great potential can be seen experimentally when rehabilitation is combined with epidural stimulation, pharmacological agents (serotonin and dopamine) and neuromodulation to induce plasticity [118, 119]. Brand et al. [120] demonstrated that voluntary control of locomotion was restored after a paralyzing spinal cord injury upon rehabilitation and extensive plasticity induction. However, this return of function could have been due to the upright posture paradigm of the rehabilitation, as opposed to being caused by the process of voluntary movement. Overall, the general paradigm of functional recovery from various forms of paralysis has been positive, with paralyzed patients beginning to move their hands [121, 122], stand [123] and take steps [124, 125]. There has been a case where a paraplegic patient who had a complete SCI for 3 years recovered stepping ability after neuromodulation of the lumbosacral spinal networks to induce plasticity and neuroregeneration [126].

The upregulation of CNS regeneration for functional recovery can also be achieved through exercise training. Brain-derived neurotrophic factor (BDNF) expression and rates of axonal sprouting are increased with treadmill training [127]. BDNF, neurotrophin-3 (NT-3) and nerve growth factor (NGF) are among the key neurotrophic factors that influence the regeneration of the nervous system. BDNF plays a role in stimulating the growth of corticospinal tract neurons, NT-3 improves the survival of those neurons, and NGF is an important trophic factor for small diameter sensory neurons. Thus, a thorough knowledge of their effects on specific neuronal populations is needed to guide effective targeting, especially when used alongside other treatment options, such as stem cell grafts and nerve bridges, to improve plasticity and neuroregeneration [128]. The utilization of these neurotrophic factors has led to functional recovery in rodents [129] and primates [130]. Yang et al. [129] developed a novel matrix scaffold made up of chitosan and slow-release NT-3, inducing endogenous neural stem cells to proliferate, migrate and differentiate into neurons while reducing posttraumatic inflammatory processes such as glial scar formation. The reduction in inflammatory processes is a key part of these treatments in neuroregeneration, as Rosenzweig et al. [131] demonstrated that the restorative effects of human stem cell grafts in primates were successful only when the inflammatory process was mitigated. In experiments conducted by Yang et al. [129], nascent neurons formed the basis of an intermediate circuit that relayed ascending and descending signals. Therefore, this regeneration of intermediate relay neurons, coupled with rehabilitation and induced plasticity, allows functional recovery of movement to sites of focal injuries, especially to those in the spinal cord.

In addition to these physiological methods of restoring functional tissue to achieve functional recovery, bioelectronic implants have been introduced that act to electronically relay messages across gaps in the nervous system. These electronic implants can target circuits located in the brain, midbrain, and spinal cord to improve motor and autonomic function. They carry out their function by augmenting the plasticity of the spared circuits and residual projections when coupled with rehabilitation training programs. This approach is especially useful when the area of damaged tissue is too large to be bridged purely by physiological means. However, this method is currently limited by the short timespan before the implant is rejected by the body’s immune system through inflammatory processes such as gliosis [132].

In humans, the sense of proprioception is considered essential for coordinated movement, but not in smaller animals such as rodents. Taking this into account, Wagner et al. [133, 134] devised a pattern-based electronic stimulation, rather than the continuous stimulation reported in previous studies, which coordinates with different phases of the gait cycle. This ensures that the descending stimulus signal is sent, while ascending proprioceptive stimulation is also given the chance to be received. This approach resulted in three individuals with SCI walking with minimal assistance that persisted post stimulation. This result suggests that patterned electronic stimulation helps to guide plasticity in the spinal cord effectively when combined with rehabilitation to provide a synergistic effect for functional recovery [135, 136]. Therefore, to be able to develop effective functional recovery from electrical stimulation combined with rehabilitation, patterned stimulation is also necessary. To develop this pattern, there is a need to understand the neurophysiological aspects of the neural communication required for movements. For locomotion, this understanding would include that of the gait cycle. However, with the upper limbs, the range of movements is significantly larger, given their greater utility. Thus, Sburlea et al. [137] explored human grasping, represented in neural, muscle and kinematic signals. On the basis of this effort, additional work can be done to elicit similar success for upper limb movements. In terms of restoring psychological, cognitive and neurological function through a similar concept, neurofeedback through real-time functional magnetic resonance imaging would be useful. Stimulus patterns can be patterned according to the neurofeedback received to generate a successful rehabilitation protocols for recovering psychological, cognitive or neurological function [138].

There has been significant interest in the utilization of robotic devices to conduct neurorehabilitation training. Robotic devices serve the useful purpose of reducing the load on therapists during ambulation and recording the biomedical gait parameters more accurately than can be accomplished with manual physical therapy [139]. However, a multicentered randomized control trial found that robot-assisted training of the upper limbs after stroke did not result in significantly better care for patients with moderate and severe upper limb functional limitations than offered by traditional therapy. Forced robotic gait training has also been implicated in altering natural patterns of muscle activation, such as reduced ankle flexion-extension and higher quadriceps-hamstring activity in the swing phase compared to ambulation training on a treadmill [140]. Therefore, robotic devices are currently good aids for therapists to use for conduct rehabilitation training but are not yet suitable as complete replacements for usual therapy. Another interesting development in rehabilitation training has been the use of virtual reality to improve functional outcomes. There have been mixed results in terms of the benefits of Virtual Reality (VR). A systematic review found that VR can lead to improved motor, psychological and cognitive functions [141]. However, another review found that there was no distinct relationship found between immersion and improvement in motor recovery [142]. A Cochrane review found that VR was not more beneficial than conventional therapy in improving upper limb function [143]. Despite a range of conflicting opinions, it is important to recognize that the development of VR is still in its infancy. Currently, VR can be used as an adjunct to usual care to increase overall therapy time. As the technology develops, further reassessments are necessary to determine its effectiveness in improving functional recovery in patients in rehabilitation programs.

In addition to the rehabilitation and the functional recovery of limb functions, plasticity and neuroregeneration with rehabilitation can benefit other areas damaged by gunshot wounds or bomb blasts, as indicated in Table 2.

**Table 2:** Rehabilitative potential for different areas of the nervous system damaged by blast or gunshot injuries

Areas for rehabilitation improvement	Affected area	Methods that can be used with observed impacts
Movement disorders in Parkinson’s Disease	Basal ganglia [144]	• Long-term deep brain stimulation of the subthalamic nuclei • Restorative effects of global structural and functional connectivity as a result of plasticity and neuroregeneration [145] • Stimulation of mesencephalic locomotor region [146] [analogous to the pedunculopontine nucleus in humans [147]
Motor recovery after stroke	Unilateral cervical contusion [148]	• Vagal nerve stimulation • Release of monoamines within cerebral cortex • Promotes plasticity of neural circuits and enhances motor learning [148, 149]. • Activity-dependent plasticity also occurs [150].
Allodynia	Mid-thoracic contusion SCI [151]	• Induces plasticity via stimulation to the nucleus raphe magnus to augment serotonin release [151].
Speech	Left fronto-temporo-parietal region (15708219)	• Intensive speech therapy [152, 153] • Combined with pharmacological therapies [154–157] • Combined with noninvasive brain stimulation [158–161]. • Results are promising, but sample sizes have been small [162].
Eating and swallowing	Motor cortex	• Sensory input essential as it drive changes in cortical circuitry [163]. • Neuromuscular stimulation induces plasticity changes [164].
Visual field and recognition	Visual cortex	• Restitutive capacity is limited [165] • Compensatory mechanism are effective – shifting the visual field border towards the hemianopic side in hemianopia to improve spatial orientation and mobility [165]. • New visual functions – enhancement of the resolution to make it greater than that of the retina [165]. • Plasticity level in higher visual functions is unknown [166]. • Plasticity through cross-mode sharing of visual pathways with tactile or auditory pathways through extensive training and practice [167].
	Optic Nerve	• Optic nerve with appropriate deletions of physiological “brakes” or additions of “facilitators” can regenerate centrally from the retinal ganglion cells [47].
Cognitive (thinking, reasoning, judgment and memory)	Frontal cortex	• NF training can lead to positive memory function and normalization of pathological brain activation patterns [168]. • Enriched environment promotes synaptic plasticity [169]. • Selective serotonin reuptake inhibitors administered acutely after brain injury may induce plasticity similar to that seen in the critical period [170]. • Normal plasticity becomes dysfunctional postinjury, failing to confer neuroprotection and to prevent further cell death. Therapies should target aspects of normal plasticity that are altered postinjury [171].
Bowel and bladder control	SCI above the sacrum	• Early sacral neuromodulation following SCI reduces the extent of secondary injury and maladaptive neural restricting [172]. • Further evidence needed to support this theory. • EGFR inhibition promotes nerve regeneration in vitro and in vivo, with bladder function restored in rodents [173].
Emotional control	Fear memories	• Inhibition of NgR1 can help with the recovery of emotional control postinjury [174, 175].

*NF* Neurofeedback, *SCI* Spinal Cord Injury, *EGFR* epidermal growth factor receptor, *NgR1* Neuronal Nogo-66 receptor 1

These integrated treatment plans provide a means to improve outcomes and recovery time following an injury that previously would have been treated only palliatively. This reduced recovery time and better recovery enable veterans suffering from injuries to return to their daily lives or even back to service. Looking beyond the current horizon, if such integrated treatment plans can work for injured individuals, it is possible to enhance the function and/or reduce the workload of currently healthy soldiers with integrated technologies [176, 177]. Leveraging our understanding of relay circuits and the plasticity of the brain, walking functions and limb movements can be translated appropriately to an external exoskeleton when proprioceptive sensory input is provided, with the result of alleviating the soldier’s workload and reducing fatigue. Furthermore, with a plastic brain, it may be possible to learn new abilities and control external devices, thus improving the functionality of a soldier.

## Conclusions

Injury to the nervous system, as seen in SCI and TBI, has been an area of concern because of its high incidence and lack of clear and effective treatment strategies. We now know the key molecular mechanisms that underlie the failure of nerve regeneration in the CNS and under conditions of chronic injuries in PNS. This knowledge has enabled us to use neuroregeneration and plasticity induction techniques to stimulate the sprouting of nascent neurons and modulate labile ones. Neurorehabilitation techniques have also been developed to incorporate our current understanding of movement, resulting in an enhanced recovery of function by guiding nascent and labile neurons into the appropriate end locations effectively. Further work needs to be done on 1) understanding the balance between excitatory and inhibitory signals, 2) determining the effect of injury on this balance and 3) identifying targets that can be used to control this balance. This level of information would enable accurate modulation of the neuronal network, leading to activation of localized plasticity while preserving stability in other areas. With regard to rehabilitation treatments, we need to understand the pattern of upper limb movements, as they have a higher degree of freedom than lower limb gaits. Applying this knowledge to develop an appropriate rehabilitative protocol while allowing for proprioceptive feedback is the next step. Another effort that would benefit the field involve developing a method to accurately send descending outputs while preserving the ascending inputs in a 2-way flow of information to replace the 1-way flow currently used.

## Data Availability

Not applicable.

## Abbreviations

AAD: Acute axonal degeneration
AD: Alzheimer’s disease
BBB: Blood-brain barrier
CNS: Central nervous system
CSPG: Chondroitin sulfate proteoglycans
EAE: Experimental autoimmune encephalomyelitis
iPS: Induced pluripotent stem (cells)
MAG: Myelin-associated glycoprotein
MS: Multiple sclerosis
NGF: Nerve growth factor
OMgp: Oligodendrocyte myelin glycoprotein
PNN: Perineuronal net
PNS: Peripheral nervous system
RAGs: Regeneration-associated genes
SCI: Spinal cord injuries
TBI: Traumatic brain injury
VR: Virtual Reality
